# Patient-Reported Experience Measures in Pediatric Healthcare—A Rapid Evidence Assessment

**DOI:** 10.1177/23743735241290481

**Published:** 2024-12-09

**Authors:** C. Bartholdson, E. Broström, M. D. Iversen, J. Granhagen Jungner

**Affiliations:** 1 Department of Women's and Children's Health, 27106 Karolinska Institutet, Stockholm, Sweden; 2 Highly Specialized Pediatric Medicine and Orthopedics, Astrid Lindgren Children's Hospital, Karolinska University Hospital, Stockholm, Sweden; 3 College of Health & Wellness, Johnson & Wales University, Providence, RI, USA; 4 Section of Clinical Sciences, Division of Rheumatology, Immunology & Immunity, Brigham & Women's Hospital, 1811 Harvard Medical School, Boston, MA, USA

**Keywords:** pediatrics, patient-reported experience measures, patient experience, review

## Abstract

This study aimed to describe what is known in the scientific literature about patient-reported experience measures (PREMs) in pediatric healthcare and identify areas for further exploration. PubMed, Web of Science, CINAHL, Google Scholar, COCHRANE, and SveMed+ combined with free text search in FireFox and Safari web browsers using Medical Subject Headings terms were used. Outcomes of interest were patient experience and measures of these constructs. Of the 316 studies identified, 68 met the inclusion criteria. Forty-eight studies (72%) were published between 2015 and 2020 and more than half (53%) were published in Europe. Most studies of PREMs in pediatric healthcare included adult proxies as participants. Seventy-eight percent of studies consisted of > 100 participants. Thirty-six studies (53%) were quantitative studies, 26 (38%) were evaluative studies of patient experience measures, and 6 (9%) were qualitative in design. Three hundred eleven domains were identified and further categorized into 14 domain areas. This research is important because it aims to amplify the voices of children in healthcare and establish a foundation for developing validated pediatric-PREMs that is grounded in children's firsthand experiences of care, rather than relying primarily on proxy accounts.

## Introduction

Patient-reported experience measures (PREMs) are essential tools in the development and improvement of healthcare systems.^1^ The most common objective of healthcare system is to maximize the health of individuals and the populations they serve, and to do so in an equitable way within budgetary parameters. The patient perspective is increasingly relevant in overcoming the demographic, epidemiological, and economic challenges faced by many healthcare systems. In the clinical setting, measuring patient-reported metrics helps to focus the healthcare interaction on the needs of the individual. In broad terms, the PREM definition is expressed as measuring patients’ interactions with healthcare systems and identifying the degree to which their needs are being met. PREMs help determine whether patients have experienced certain care processes rather than focus solely on their satisfaction with the care received (which may be subject to bias).^2^

Although the patient's experience in healthcare is an important factor in the quality and development of care, there is little knowledge about patient and family experiences of pediatric healthcare.^3^ To measure patient's experiences in pediatric healthcare, clinics have traditionally created and implemented their own surveys covering different dimensions of care such as overall impression, emotional support, participation and involvement, respect and responsiveness, continuity and coordination, information shared and patients’ understanding of this information, as well as accessibility. Thus, minor efforts have been made to measure patient experiences in pediatric healthcare using valid and reliable instruments.^4^ However, in Sweden, Nordlind et al recently translated, culturally adapted and validated a PREM for children based on a PREM from the United Kingdom.^5^ The authors identified the significance of a thorough procedure of adaptation and validation to guarantee excellence and relevance for children accessing healthcare in various contexts and nations.^5^ A similar study has been carried out in Canada where the authors developed an adapted rendition of the initial instrument, preserving its fundamental concept while adjusting to fit the cultural context of Canada.^6^

The scarcity of validated and reliability-tested PREM instruments in pediatric healthcare highlights a significant gap in the research field. This gap underscores the importance of identifying existing PREMs and areas where further research and development are needed. Thus, the aims of this rapid evidence assessment (REA) were to describe what is known in the scientific literature about PREMs in pediatric healthcare, and to identify areas for further exploration. The specific aims were to investigate:

- in what pediatric healthcare settings are PREM used?
- the most common respondents of PREM questionnaires; children, adolescents, and/or parents/caregivers (proxy)?
- what methods are used to collect data about PREMs in pediatric healthcare?
- what domains are most cited in the existing literature?

## Method

We conducted this REA review according to the Center for Evidence-Based Medicine guidelines.^7^ A REA review is a systematic methodology that provides a clear and structured approach to identifying what is known and what is not known in the scientific literature^8^ focusing on, in our case, PREMs in pediatric healthcare. The REA review was used because it allows a focused review on specific questions or issues in a phenomenon, providing targeted insights without the extensive breadth of a full systematic review. The REA checklist was used as the reporting guideline for this study.^7^ To optimize the initial search strategy, we used the Sample, Phenomenon of interest, Design, Evaluation, Research search tool,^9^ to identify relevant issues and questions in relation to our aim. The sample included pediatric patients and/or their parents/caregivers (proxy). The age range for pediatric patients was set to 0 to 18 years but was not limited to that in studies where young adults were described in the same cohort. Phenomena of interest were PREMs, patient satisfaction, and patient experience. Both qualitative- and quantitative designs were included. Evaluation measures included experiences, satisfaction, attitudes, and perceptions. Questions about research types included instrument- and questionnaire development, evaluation- and implementation research, and interview studies. One of the features of a REA that distinguishes a REA from a traditional review is the prespecification of criteria for including and excluding studies.^7^ This systematic and transparent approach to study selection provides a more rigorous and reliable synthesis of evidence compared to traditional literature reviews.

### Search Strategy

A literature search was performed on all peer-reviewed articles published between January 2010 and December 2021. Given the rapid nature of REAs, including a timeframe helped streamline the search and selection process, saved time and resources while still provided valuable insights. The inclusion criteria were as follows: articles published in English or Nordic languages; quantitative and qualitative studies that described the development of various patient-reported surveys measuring patient reported experiences; assessed PREM outcomes within pediatric healthcare, including both out- and inpatient care. The exclusion criteria were articles reporting on pediatric Patient Reported Outcome Measures; articles not published in English or Nordic languages; intervention studies and literature reviews. The reason for excluding intervention studies was the focus on PREM in pediatrics as a phenomenon rather than evaluating the experience of interventions. The reason for excluding reviews was that using primary studies ensured consistent inclusion criteria and data extraction methods, which would have been harder to achieve if we would have integrated other reviews with varying methodologies.

The following 6 databases were used to identify peer-reviewed publications: PubMed, Web of Science, CINAHL, Google Scholar, COCHRANE, and SveMed+. This process, together with free text FireFox and Safari web browser searches, was used to find literature and additional studies related to the topic. An experienced research librarian at the University Library provided advice on the search terms list. Due to the subject's elusiveness (a steady and consistent vocabulary does not exist), the search strategy had to be both narrow and broad allowing an openness to finding new keywords and search terms. For example, “experiences of care and patient reported experiences and PREM” is expressed in many ways and the terms are not indexed into Medical Subject Headings (MeSH) terms.

**Figure 1.**
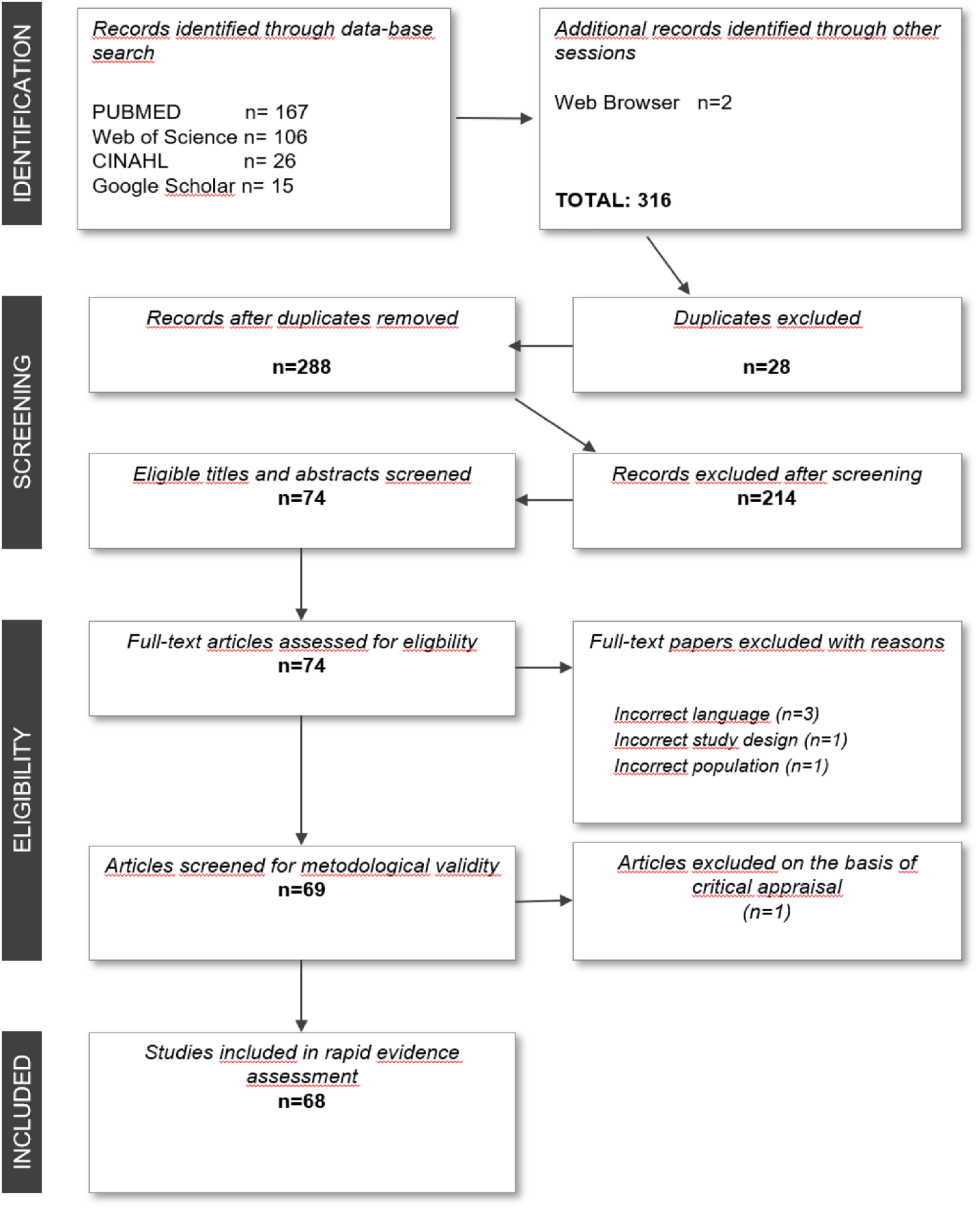
The PRISMA-SAR screening and selection process for studies of PREMs in pediatric healthcare including.

PRISMA: Preferred Reporting Items for Systematic Reviews and Meta-Analysis; PREMs: patient-reported experience measures.

Initially, different search blocks were constructed to invent different search possibilities. The search blocks consisted of a combination of MeSH terms and of free-text keywords. These terms were then combined using a Boolean search technique. Search blocks were combined with “AND.” The subject/free-text words within the blocks were combined with “OR.” An example of a search using MeSH terms and subject words/free-text words is: (“Patient Reported Outcome Measures” OR “Patient Satisfaction” OR “Attitude to Health” OR “Patient-reported experience measures” OR “Patient reported experience measures” OR “Patient Centered Outcomes Research” OR “Patient Outcomes Assessment”) AND (Survey* and Questionnaire* OR Interview*) AND (Adolescent* OR Child* OR Teen* OR Youth*) (Figure 1).

Since the purpose of this REA was to obtain a broad search within the subject area, the search began with identifying key articles that were relevant to the research purpose and questions. These articles served as a foundational set of references. If the key articles were not included, the search strategy was modified. This is a common practice in systematic reviews, as key articles provide valuable insights into relevant search terms and resources. The Preferred Reporting Items for Systematic Reviews and Meta-Analysis^10^ guidelines governed the conduct and reporting of this review.

Three researchers screened and selected titles and abstracts against the predefined inclusion criteria. Next, two researchers independently screened selected full text articles. In both steps, conflicts were resolved through discussion and consensus. Following identification of the full text studies, three researchers independently extracted the data from articles based on the following parameters: Country, Setting, Aim, Patient population age (N), Proxy (N), Response rate, Methods, and Domains.

## Results

### Search Process

The database search process yielded 314 studies plus two studies identified from the web-based search, for a total of 316 potential studies. Of the 74 articles included after title and abstract screening, six articles were excluded for the following reasons: three were published in other languages (Hungarian^6^, Spanish^56^, and Polish^61^), one study included mostly young adults aged 16 to 25 years,^25^ one paper was a review^16^ and one was a conference abstract.^43^ Thus, 68 studies are included in this review. The 68 result articles are shown in Table 1.

**Table 1.** Result References—Included in the Rapid Evidence Assessment.

No.	Author(s)	Year	Title	Journal	Volume	Issue	Page(s)
1	No reference since it is the header line in the excel data-set						
2	Ademuyiwa et al	2017	Assessment of parents’ satisfaction with paediatric surgery services at a tertiary hospital in South West Nigeria: a quality control check	*Ann Med Health Sci Res*	7	1	42-46
3	Ahmed et al	2017	Evaluation of patient satisfaction in pediatric dermatology	*Pediatr Dermatol*	34	6	668-672
4	Alazmah et al	2021	Developing a child patient satisfaction survey: a quality improvement project	*Eur Arch Pediatr Dent*	22		209-217
5	Bal et al	2020	Patient reported experience in a pediatric emergency department	*J Patient Exp*	7	1	116-123
6	Article excluded						
7	Bitzer et al	2012	Patient satisfaction in pediatric outpatient settings from the parents’ perspective-The Child ZAP: A psychometrically validated standardized questionnaire	*BMC Health Serv Res*	12	1	1-1
8	Boss and Thompson	2012	Patient experience in outpatient pediatric otolaryngology	*Laryngoscope*	122	10	2304-2310
9	Brenn et al	2016	Outpatient outcomes and satisfaction in pediatric population: data from the postoperative phone call	*Pediatr Anaesth*	26	2	158-163
10	Brown et al	2014	Satisfaction in child and adolescent mental health services: Translating users’ feedback into measurement	*Adm and Policy in Mental health services research*	41		434-446
11	Bruyneel et al	2017	Validation of the Child HCAHPS survey to measure pediatric inpatient experience of care in Flanders	*Eur J Pediatr*	176		935-945
12	Byczkowski, Kollar, and Britto	2010	Family experiences with outpatient care: do adolescents and parents have the same perceptions?	*J Adolesc Health*	47	1	92-98
13	Byczkowski, Fitzgerald, et al	2013	A comprehensive view of parental satisfaction with pediatric emergency department visits	*Ann Emerg Med*	62	4	340-350
14	Chamla et al	2016	Caregiver satisfaction with paediatric HIV treatment and care in Nigeria and equity implications for children living with HIV	*AIDS care*	26		153-160
15	Clark et al	2019	Children's drawings with narratives in the hospital setting: Insights into the patient experience	*Hosp Pediatr*	9	7	495-500
16	Article excluded						
17	Coleman et al	2020	The child's voice in satisfaction with hospital care	*J Pediatr Nurs*	50		113-120
18	Dackiewicz et al	2016	Patient experience assessment in pediatric hospitals in Argentina	*Int J Qual Health Care*	28	6	675-681
19	Ehwerhemuepha et al	2017	Impact of anesthesiologists on parental perioperative satisfaction scores	*Pediatr Anaesth*	27	9	949-954
20	Ellemunter et al	2015	Evaluating patient experience in a cystic fibrosis centre using a disease-specific patient satisfaction questionnaire	*Eur J Pediatr*	174		1451-1460
21	Ellzey et al	2015	Parent perceptions of quality of life and healthcare satisfaction for children with medical complexity	*J Pediatr Rehabil Med*	8	2	97-104
22	Espinel et al	2014	What parents say about their child's surgeon: parent-reported experiences with pediatric surgical physicians	*JAMA Otolaryngol Head Neck Surg*	140	5	397-402
23	Furness et al	2017	Cancer patient experience in the teenage young adult population—key issues and trends over time: an analysis of the United Kingdom National Cancer Patient Experience Surveys 2010-2014	*J Adolesc Young Adult Oncol*	6	3	450-458
24	Fustino et al	2019	Improving patient experience of care providers in a multispecialty ambulatory pediatrics practice	*Clin Pediatr (Phila)*	58	1	50-59
25	Article excluded						
26	Girling et al	2015	What young people want from their diabetes team: developing a patient reported experience measure (PREM) for young people with type 1 diabetes	*Pract Diab*	32	4	142-147
27	Article excluded						
28	Gómez-de-Terreros-Guardiola et al	2019	A Measurement Scale to Assess Children's Satisfaction with Hospitalization in the Andalusian Population	*Int J Environ Res Public Health*	16	17	3110
29	Gore et al	2016	New patient-reported experience measure for children with allergic disease: development, validation and results from integrated care	*Arch Dis Child*	101	10	935-943
30	Grandjean et al	2017	Measurement of parent satisfaction in the paediatric intensive care unit–Translation, cultural adaptation and psychometric equivalence for the French-speaking version of the EMPATHIC-65 questionnaire	*Intensive Crit Care Nurs*	38		40-45
31	Hagen et al	2019	Parental satisfaction with neonatal intensive care units: a quantitative cross-sectional study	*BMC Health Serv Res*	19		1-2
32	Halleran et al	2019	Development of a patient-reported experience and outcome measures in pediatric patients undergoing bowel management for constipation and fecal incontinence	*J Pediatr Gastroenterol Nutr*	69	2	34-38
33	Harder et al	2016	Effects of patient-centered medical home transformation on child patient experience	*J Am Board Fam Med*	29	1	60-68
34	Hargreaves and Viner	2012	Children's and young people's experience of the National Health Service in England: a review of national surveys 2001-2011	*Arch Dis Child*	97	7	661-666
35	Hargreaves, McDonagh, and Viner	2013	Validation of You're Welcome quality criteria for adolescent health services using data from national inpatient surveys in England	*J Adolesc Health*	52	1	50-57
36	Hodgkinson et al	2019	Development and pilot of a burns-specific patient-reported experience measure	*Burns*	45	7	1600-1604
37	Iversen et al	2019	The adolescent patient experiences of diabetes care questionnaire (APEQ-DC): reliability and validity in a study based on data from the Norwegian childhood diabetes registry	*Patient Relat Outcome Meas*	27		405-416
38	Janhunen et al	2019	Quality of pediatric emergency care as assessed by children and their parents	*J Nurs Care Qual*	34	2	180-184
39	Kaipio et al	2019	Development of the Patient Experience Questionnaire for Parents of Pediatric Patients (PEQP)	*InITCH*	1		200-205
40	Kanter et al	2020	Perceptions of US adolescents and adults with sickle cell disease on their quality of care	*JAMA Netw Open*	3	5	
41	Kapp et al	2017	Identifying the determinants of perceived quality in outpatient child and adolescent mental health services from the perspectives of parents and patients	*Eur Child Adolesc Psychiatry*	26		1269-1277
42	Ahmed et al	2020	Identifying areas for improvement in paediatric inpatient care using the Child HCAHPS survey	*Paediatr Child Health*	25	6	365-371
43	Article excluded						
44	Knapp et al	2014	Assessing patient experiences in the pediatric patient-centered medical home: a comparison of two instruments	*Matern Child Health J*	18		2124-2133
45	Laos et al	2012	Mobile pediatric emergency response team: patient satisfaction during the novel H1N1 influenza outbreak	*Acad Emerg Med*	19	3	274-279
46	Latour et al	2011	Construction and psychometric testing of the EMPATHIC questionnaire measuring parent satisfaction in the pediatric intensive care unit	*Intensive Care Med*	37		310-318
47	Lew et al	2010	Factors affecting parental satisfaction following pediatric procedural sedation	*J Clin Anesth*	22	1	29-34
48	Lowe et al	2012	Predictors of parent satisfaction in pediatric laceration repair	*Acad Emerg Med*	19	10	1166-1172
49	Lunt et al	2020	Validation of novel patient-centered juvenile idiopathic arthritis-specific patient-reported outcome and experience measures (PROMs/PREMs)	*Pediatr Rheumatol*	18	1	1-9
50	Lygre et al	2020	How can we improve specialist health services for children with multi-referrals? Parent reported experience	*BMC Health Serv Res*	20	1	1-1
51	Maini et al	2018	Evaluation of a questionnaire to measure parent/carer and child/young person experience of NHS epilepsy services	*Seizure*	63		71-78
52	Margaritis et al	2012	Exceeding parents’ expectations in Ear–Nose–Throat outpatient facilities: The development and analysis of a questionnaire	*Eval Program Plann*	35	2	246-255
53	Matziou et al	2011	Parents' satisfaction concerning their child's hospital care	*Jpn J Nurs Sci*	8	2	163-173
54	Nguyen et al	2020	Parental satisfaction with quality of neonatal care in different level hospitals: evidence from Vietnam	*BMC Health Serv Res*	20		1-9
55	Parra et al	2017	Patient experience in the pediatric emergency department: do parents and children feel the same?	*Eur J Pediatr*	176		163-167
56	Article excluded						
57	Rubenstein et al	2016	Pediatric interventional radiology clinic–how are we doing?	*Pediatr Radiol*	46		1165-1172
58	Sadlo et al	2014	Measuring satisfaction with health care in young persons with inflammatory bowel disease-an instrument development and validation study	*BMC Health Serv Res*	14		1-0
59	Salmani et al	2015	The process of satisfaction with nursing care in parents of hospitalized children: a grounded theory study	*Int J Pediatr*	3		
60	Simmons et al	2014	Development of a satisfaction scale for young people attending youth mental health services	*Early Interv Psychiatry*	8	4	382-386
61	Article excluded						
62	Solheim and Garratt	2013	Parent experiences of inpatient pediatric care in relation to health care delivery and sociodemographic characteristics: results of a Norwegian national survey	*BMC Health Serv Res*	13	1	1-2
63	Tan et al	2014	A survey of children's reported experience in outpatient pediatric ophthalmology clinics	*J Pediatr Ophthalmol Strabismus*	51	5	270-273
64	Toomey et al	2017	Variation in family experience of pediatric inpatient care as measured by child HCAHPS	*Pediatrics*	139	4	
65	Toomey et al	2015	The development of a pediatric inpatient experience of care measure: Child HCAHPS^®^	*Pediatrics*	136	2	360-369
66	Urben et al	2015	Patients' satisfaction with community treatment: a pilot cross-sectional survey adopting multiple perspectives	*J Psychiatr Ment Health Nurs*	22	9	680-687
67	Wangmo et al	2016	Parents' and patients' experiences with paediatric oncology care in Switzerland-satisfaction and some hurdles	*Swiss Med Wkly*	146		
68	Waseem et al	2018	Parental satisfaction with being present in the operating room during the induction of anesthesia prior to pediatric neurosurgical intervention: a qualitative analysis	*Journal of Neurosurgery: Pediatrics*	21	5	528-534
69	Williams et al	2018	A UK survey of the experience of service provision for children and young people with epilepsy	*Seizure*	60		80-85
70	Wood et al	2018	Eliciting the experiences of the adolescent-parent dyad following critical care admission: a pilot study	*Eur J Pediatr*	177		747-752
71	Wray and Oldham	2020	Using parent-reported experience measures as quality improvement tools in paediatric cardiothoracic services: making it happen	*Int J Qual Health Care*	32	2	140-148
72	Wray, Hobden, et al	2018	Hearing the voices of children and young people to develop and test a patient-reported experience measure in a specialist paediatric setting	*Arch Dis Child*	103	3	272-279
73	Wray et al	2018	Parents’ experiences of caring for their child at the time of discharge after cardiac surgery and during the postdischarge period: Qualitative study using an online forum	*J Med Internet Res*	20	5	155
74	Ye et al	2016	One size does not fit all: pediatric patient satisfaction within an integrated health network	*Am J Med Qual*	31	6	559-567
75	Ziniel et al	2016	Development and psychometric characteristics of the pediatric inpatient experience survey (PIES)	*Int J Qual Health Care*	28	2	191-199
76	Züllich et al	2012	Migration background and patient satisfaction in a pediatric nephrology outpatient clinic	*Pediatr Nephrol*	27		1309-1316

### Publication Period, Country, and Setting of Study

Forty-eight studies (72%) were published between 2015 and 2020. Most studies of PREMs in pediatric healthcare were published in Europe (53%), followed by North/South America (38%) (Supplemental Table S1). With respect to study settings, most published studies were conducted in outpatient care facilities located within a hospital. The remaining studies were conducted in emergency departments and pediatric intensive care units, inpatient and pre/postoperative care units. Some questionnaires were distributed to assess care at the hospital level, for example, all outpatient clinics at a pediatric hospital. Furthermore, PREMs were provided to assess care by diagnosis, type of health center, for use in combined health services and within patient registries (Table 2).

**Table 2.** Settings in which studies of PREMs in pediatric healthcare were conducted (*n* = 67, 1 missing).

**Hospital-level (*n* = 7)**			
Multiclinic children's hospital			
General pediatric, onco/haematology, nephrology, surgery, and internal medicine			
Children's hospital			
Outpatient clinics			
Pediatric and surgical units			
Neonatal care (2 hospitals, national/provincial))			
Pediatric in-and out pat settings			
**Differentiation within the hospital (*n* = 43)**			
**Outpatient (*n* = 18)**	**ED/PICU (*n* = 11)**	**Inpatient (*n* = 9)**	**Post/pre OP (*n* = 5)**
Dermatology (1)	Pediatric ED (6)	Pediatric (8)	Surgery (2)
Dental (1)	PICU (4)	Urban medical center (1)	Perioperative (1)
Ambulatory (1)	NICU (1)		Sedation service (1)
Otolaryngology (2)			Pre OP (1)
Mental health (2)			
Teen health center (1)			
HIV (1)			
Surgery (1)			
CF (1)			
Intensive outpatient care (1)			
Diabetes (2)			
Rheumatology (1)			
Radiology (1)			
Ophthalmology (1)			
Nephrology (1)			
**Diagnosis level diff. health services (*n* = 6)**		**Combined health services (*n* = 1)**	
Cancer (2)		Primary care, outpatient specialty care, ED, inpatient care (1)	
Allergy (1)			
Sickle cell decease (1)			
Epilepsy (1)			
Cardio-thorax (1)			
**Health centers (*n* = 5)**		**NHS/register (*n* = 5)**	
Pediatric family medicine (1)		Adolescent health services (1)	
Patient-centered medical home (1)		Inpatient services (1)	
Mental health services (3)		Burn services (1)	
		Epilepsy services (1)	
		IBD (1)	

PREMs: patient-reported experience measures.

### Sample Sizes and Sample Characteristics

Most studies of PREMs in pediatric healthcare included adult proxies as participants; followed by studies of children + proxies and children. Half of the articles included between 101 and 1000 participants, and 28% of studies included more than 1000 participants. This means that 78% of the articles consisted of more than 100 participants. Within studies that only included children, adolescents and/or young adults (*n* = 11), the age range were between 2 and 25 years. Among studies that included children and their proxy (*n* = 17), the age of children/adolescents ranged from newborn to 19 years. Three articles, including children and children+proxy did not report ages (Table 3).

**Table 3.** Participants in studies of PREMS in pediatric healthcare.

Participants (*n*)	Number* unknown	<50	51-100	101-1000	>1000	Number of articles (*N* = 68)
Children		1	2	5	4	12/68 (18%)
Children + proxy^1,2,3^	1	4	2	7	5	19/68 (26%)
Proxies		2	1	22	10	35/68 (51%)
Total	1 (2%)	7 (10%)	5 (7%)	34 (50%)	19 (28%)	
Missing						2/68 (3%)

PREMs: patient-reported experience measures.

1. Number of participants unknown.

2. Two articles define total number of participants, but the division between children and proxy is unknown.

3. One article defines number of proxies but not number of children.

### Response Rate

In total, the study included 68 articles, where 11 included children and 56 included children+proxies and proxies. In three of 11 articles, the response rate for children was reported (1%, 59%, and 89%). In 32/56 articles response rate for children+proxies and proxies were reported. The range was 18% to 100%, median of response rate was 71%, and mean response rate 68%.

### Study Designs, Methods, and Aims

Among the 68 included studies, 36 (53%) were quantitative studies, 26 (38%) were evaluative studies of a PREM, and 6 (9%) were qualitative in design. The study aims in the 36 quantitative studies included the following active verbs, To: examine,^(2,76)^ determine,^(3,21,42,45)^ describe,^(5,8,20,21,38,55)^ understand,^(9,14,13)^ compare,^(34)^ identify,^(19,22,57)^ define,^(23,48)^ improve,^(24)^ explore,^(12,31,41,50,53)^ investigate/monitor,^(47,63,69)^ evaluate,^(33,62)^ measure,^(64)^ survey,^(69)^ and assess,^(40,66,54,74)^ child and parent experience. Data collection methodology in the quantitative studies included: questionnaire-based surveys,^(2,3,5,8,14,19,20,21,22,24,31,33,38,40,41)^ telephone surveys,^(9,12,13,42,45,47,48,50,53,55,57,62,63,64,66,69,74,76)^ register survey,^(23)^ meta-analysis of survey data,^(34)^ and use of a face-to-face structured survey.^(54)^

When new PREMs were being developed (*n* = 26/68, 38%) the aims of the studies included to: develop,^4,26,32,39,52,58,60,65,71^ develop and validate,^7,29^ create,^18,36^ obtain a valid scale,^28^ translate and culturally adapt and to test the PREMs psychometric equivalence,^30^construct and test reliability and validity,^46^ develop and test the survey.^72^ When already established PREM questionnaires were quality assessed, the aims of these studies were to: establish the PREMs’ psychometric properties,^10^ test feasibility and acceptability,^17^ determine the data quality, assess validity and internal consistency reliability,^37^ compare the validity and reliability of 2 PREM questionnaires,^44^ validate and pilot test,^49^ validate,^51^ study the psychometric properties,^75^ field-test,^11^ investigates the relationship between two PREM questionnaires.^35^

In six qualitative studies,^15,59,67,68,70,73^ three studies aimed to explore and understand patient and parent experiences and satisfaction with healthcare.^15,59,67^ Moreover, two of the aims identified factors important to patients and parents during their care and aimed to elicit parental experiences with care.^70,73^ Data collection methodology in the qualitative studies included: face-to-face interviews and interviews based on drawings as well as a closed online discussion group.^15,59,67, 68,70,73^

### Domains and Themes Investigated

Domains and themes were identified in the studies and subsequently, analyzed and categorized into different domain areas, based on similarity in content. A total of 311 domains were identified in the 68 studies. Those 311 domains were categorized into 14 domain areas and named and ranked according to their frequencies. The domain areas were healthcare professionals, healthcare organization, overall satisfaction, information/knowledge, care treatment and tests, communication, environment, approach and attitude, participation/involvement in care, access to healthcare, support with emotions and feelings, pain, well-being, and other. Each domain area is presented in Table 4.

**Table 4.** Presentation of Domain Areas and Content.

Domain area	Content	Examples of domains
Healthcare professional (*n* = 43)	This domain area pertained to parent and child experiences and satisfaction with healthcare professionals (HCPs) working in hospital or outpatient settings. More specifically, this domain described features such as knowledge of medical history, medical skills, the care provided and interdisciplinary collaboration. The domains also addressed perceptions of how busy HCPs were, kindness displayed by HCPs and HCPs’ ability to provide trust.	“Satisfaction with nursing care” and “interpersonal skills of staff”
Organization (*n* = 39)	This domain area focused on the experiences and satisfaction with the coordination of the patient journey throughout the hospital experience. The areas covered on this healthcare journey included accessibility of HCPs, process of making appointments, arrival and registration experience, appointment and inpatient length of stay, frequency of sessions, diagnosis, waiting time, time available for questions, discharge process, and return to life at home:	“Coordination of care” and “admission and discharge and home care preparation”
Overall satisfaction (*n* = 36)	This domain area included general impressions of care based on overall assessments. The domain-area also involved perceived quality of medical care, the degree to which parents’ expectations were fulfilled and a promised service was performed accurately. Moreover, overall satisfaction included whether parents had “faith” in the healthcare provider and were willing to seek continued care or recommend the healthcare provider to others	“Best and worst things” and “overall rating of care”
Information and knowledge (*n* = 29)	This domain area included the quantity of information shared with the child and parent and whether that information was understandable. Different aspects of information were covered such as: the child's state of health, daily routines, relevance of information provided, discharge process, and examinations and tests. The knowledge in this domain-area included: learning about new things from multiple HCPs and information leaflets as well as the ability to assess and understand the clinical test results.	“Information/education” and “learning new things from multiple members of staff and leaflets”
Environment (*n* = 26)	This domain area addressed various clinical setting factors such as privacy, confidentiality, hygiene, waiting room comfort and tranquility that influence the satisfaction with the healthcare experience.	“When talking with doctors, nurses, and other providers” and “cleanliness of hospital room”
Care, treatment, and tests (*n* = 24)	This domain-area included proxy and child experiences and satisfaction with care, treatments received and tests. Domains in this domain-area were predominantly homogeneous and were similarly described.	“Care and treatment” and “treatment and tests”
Communication domain area (n=24)	This domain area included experiences of and satisfaction with communications among different stakeholders. The most common was communication with the child and with parents/caregivers. Moreover, the most frequent communication domains were quality of communication and specific communication about medications.	“Communication with child” and “how well doctors communicate with your child”
Approach and attitude (*n* = 22)	This domain-area involved experiences and satisfaction related the amount of respect healthcare providers showed to the family and child, whether the providers treated patients with dignity and provided family-centered care. Additionally, this domain-area included aspects of professional attitude such as recognition of and attention to patient's needs and the ability of the healthcare provider to help patients and families feel comfortable.	“Treated patient with respect and dignity” and “helping your child feel comfortable”
Participation/involvement (*n* = 21)	This domain area described experiences and satisfaction with children's and parent's decision-making and levels of engagement in the child's treatment and care, the child's readiness for procedures, and parents’ readiness to assume the care of their child at home.	“Involved in decisions about care” and “before child's procedure”
Access to care (*n* = 20)	This domain area covered the waiting time until the first appointment, healthcare provider accessibility by phone, receiving a timely follow-up appointment, getting needed referrals and the ease of contacting clinical staff without a clinic appointment.	“Getting timely appointments” and “accessibility by phone”
Support with emotions and feelings domain-area (*n*=16)	This domain area included experiences and satisfaction with support for personal issues, parental anxiety, confidence in recovery and extreme emotions (anger, fear, sadness, uncertainty). It also covered healthcare providers’ support of parent's effort to take care of the child's health.	“Emotional satisfaction” and “further support from the center staff”
Well-being (*n* = 5)	This domain area related to the child's health and safety and the child's growth and development	“Child's health and safety” and “attention to safety and comfort”
Pain (*n* = 3)	This domain-area was seldom mentioned. However, the few individuals who mentioned pain discussed pain management and how the healthcare team addressed child's pain.	“Pain management” and “paying attention to your child’s pain”
Other (*n* = 3)	This domain area constituted topics that didn't fit into any other domain-area.	“Identification of attending physician” and “condition specific questions”

## Discussion

### Synthesis - Key Findings From the Study

The results of the present REA review show that PREMs are essential tools for assessing the quality of healthcare services from the perspective of children. The interest in investigating PREMs has grown in recent years particularly in European countries, however there were lack of studies in Asia, Africa, and Oceania. Furthermore, studies on pediatric programs, were often conducted in outpatient clinics and the results were typically reported by proxy, such as parents or caregivers rather than directly by children. PREMs were generally collected through surveys, questionnaires or qualitative interviews and were tailored to specific settings or aspects of pediatric healthcare. Numerous different domains were identified in the included studies (*n* = 311). The domains were categorized into 14 domain areas similar in content. The most common domain was “healthcare professionals.” This REA has highlighted two important issues for future studies. Firstly, it has been demonstrated that pediatric PREMs most often are not developed based on children's views of what aspects of their experience with care that should be assessed, proxies are most commonly used to ascertain this information. Future pediatric PREMs research should focus on gathering data from children and co-creating PREMs with them. Secondly, researchers need to recognize the impact of culture and societal roles of children when assessing and developing pediatric PREMs to include a global perspective of children's experiences of care.

### Discussion on Findings

Giving voices to children and adolescents in matters concerning themselves is not only in line with the United Nations Convention on the Rights of the Child (CRC), and the best interests of the child, but is essential for developing person-centered pediatric healthcare. Data analysis generated through this REA revealed that almost all studies exploring PREMs in pediatric care originated from the European countries and the United States of America, thus the western world. These finding were also reported in a systematic review by Bele et al.^11^ The increased focus on children's rights and their role as coparticipants in their own care has likely contributed to this finding. This change in perspective aligns with the broader societal shifts toward recognizing and respecting the autonomy and agency of children. Children are becoming increasingly aware of their rights as the views of society shift. In a study conducted by af Ursin and Haanpää (2018), the authors reported that children's awareness of their rights varied significantly between countries.^12^ For example, children in Norway were more likely to be aware of the CRC^13^ than children from countries such as South Korea.^12^ Interestingly, Colombia stood out by being among the top two countries where children were most aware of the CRC.^12^ These results, suggest that variations exist between countries on children's knowledge of their rights with respect to healthcare delivery.

Proxies were predominantly used when exploring experiences of pediatric care. This approach was most likely due to the challenge of obtaining reliable responses from young children and the fact that pediatric PREMs have been developed based on adult preferences in what care experiences that are important to measure. We posit that the development of questionnaires measuring children's own experiences of care have not been conducted at a pace consistent with the changing views of society regarding children's participation in healthcare decision-making. Little is known about what children perceive and value regarding their experiences with healthcare. Children's views regarding the importance of specific elements of the care experience may differ. However, results from a study developing a pediatric PREM, based on children's perspectives on what aspects of their care experience that is important to measure,^14^ show that children perceive “treatment and tests,” “facilities,” and “people working at the hospital” as most important to assess.^14^ Despite this good initiative, there is a need for more studies to focus solely on the children's experience reported by the children themselves.

Our results demonstrated that more than half of the studies were quantitative, and some studies were qualitative in design. The presence of qualitative designs is not surprising as pediatric PREMs are novel and an evolving area of research. Studies using qualitative interviews with children about their care experience would most likely benefit from the possibility to ask follow-up questions and explore more deeply what the children meant when mentioning different aspects of care. We would argue that it is important to develop, and use validated pediatric PREM-questionnaires to make sure that children respond to what matters to them when sharing their experience of care. A future scooping review could focus on psychometric properties of pediatric PREMs to address the importance of validated and reliable PREM instruments. Nonetheless, we believe that combining methodological designs is well justified, as the differences in data types are likely to complement each other. However, we emphasize the importance of using the same methodology within the same hospital to enable comparisons between units.

From the results, it is clear that there are no consensuses regarding domains in pediatric PREMs. Despite the lack of consensus on core domains, the literature suggests that the domains described in pediatric PREMs aimed to capture similar content. The most common domain was “healthcare professionals.” This result ties well with a recent study, conducted in pediatric oncology, about children's perspectives on their important values in care.^15^ Focus group interviews revealed that children stressed the importance of having a personal connection with healthcare professionals during their hospitalization. The children found that the approach and emotional engagement of the healthcare professionals significantly influenced their care experience, particularly among older children.^15^ The second most common domain was “healthcare organization.” We consider that this is a result of pediatric PREMs targeting adult proxies as one might find it unlikely that children put emphasis on organizational aspects when assessing their experience with care, especially younger children. This suggests that the type of domains is most likely to change when PREMs are based on children's views of what is important to measure in terms of their experience of care. It is highly possible that, except from “healthcare professionals,” “treatment” and “care procedures” will be important domains. However, further research is needed to establish a standardized set of core domains for pediatric PREMs.

As a final remark we would like to highlight that present PREMs are often rooted to objective events that include perspectives from different stakeholders.^16^Although there are strong arguments for strengthening the child's voice about their experiences of care, it is still important that parents’ experiences are sought after. Pediatric healthcare consists of a triad of stakeholders (the child, the parents, and the healthcare professionals) who all form a very important triangulation in achieving the highest quality of care.

## Clinical Implications

Based on the findings of this REA review we recommend clinics to review their current PREMs assessing their appropriateness for being used by children. When implementing new PREMs clinics should consider using existing validated pediatric PREMs and co-create the implementation together with children.

## Limitations

A primary potential limitation of this study is the challenge of searching scientific databases and web-browsers for the subject heading, as the topic is elusive. The elusiveness of the topic is due to the absence of a steady and consistent vocabulary. The topic of “healthcare experiences, patient reported experiences and PREMs” is expressed in several ways and the terms are not indexed into MESH terms. To conduct the “rapid” review, concessions were made in the breadth and depth of the search process. Consequently, some relevant studies may have been missed. A second limitation of the study concerns the critical appraisal of included studies. A third limitation relates to the fact that the evidence on several moderators of the care experience is often based on a limited number of studies. While some studies were well controlled or randomized, the studies and methods used are too variable to draw firm conclusions. Strengths of this REA review includes the use of experienced researchers who have years of clinical experience in pediatrics, the focus on aggregation of data across various types of study designs, a systematic approach to the identification of domains with adjudication of domain decisions by an independent person.

## Conclusion

This study aimed to describe what is known in the scientific literature about PREMs in pediatric healthcare, and to identify areas for further exploration. Studies of PREMs in pediatric healthcare vary in design, and predominantly focus on proxy assessment. In pediatric care, these measures mostly include assessments of the setting, healthcare system, and provider characteristics and focus less on issues related to provision of information, ability for the family to care for the child (at the hospital/home) and the child's pain experience. This information can guide future research efforts in pediatric healthcare, helping to fill gaps in the understanding of patient experiences and the development of appropriate measurement tools to assess quality of care. This review also suggests that additional research is needed to further assess the usability and usefulness of future PREM questionnaires in pediatric healthcare.

## Supplemental Material

sj-docx-1-jpx-10.1177_23743735241290481 - Supplemental material for Patient-Reported Experience Measures in Pediatric Healthcare—A Rapid Evidence Assessment Supplemental material, sj-docx-1-jpx-10.1177_23743735241290481 for Patient-Reported Experience Measures in Pediatric Healthcare—A Rapid Evidence Assessment by C. Bartholdson, E. Broström, M. D. Iversen and J. Granhagen Jungner in Journal of Patient Experience

sj-docx-2-jpx-10.1177_23743735241290481 - Supplemental material for Patient-Reported Experience Measures in Pediatric Healthcare—A Rapid Evidence Assessment Supplemental material, sj-docx-2-jpx-10.1177_23743735241290481 for Patient-Reported Experience Measures in Pediatric Healthcare—A Rapid Evidence Assessment by C. Bartholdson, E. Broström, M. D. Iversen and J. Granhagen Jungner in Journal of Patient Experience
