# Supplementary material for: Patient-Reported Experience Measures in Pediatric Healthcare—A Rapid Evidence Assessment
Source: J Patient Exp. 2024 Dec 9;11:23743735241290481. doi: 10.1177/23743735241290481 (PMC11629420; doi:10.1177/23743735241290481)
Supplement: sj-docx-1-jpx-10.1177_23743735241290481 - Supplemental material for Patient-Reported Experience Measures in Pediatric Healthcare—A Rapid Evidence Assessment [file sj-docx-1-jpx-10.1177_23743735241290481.docx]

**Table 1 Supplementary: Countries in which studies of PREMs in pediatric healthcare were conducted (n=67, 1 missing)**

| **Europe**  **(n=36)** | **North/South America (n=26)** | **Asia**  **(n=2)** | **Africa**  **(n=2)** | **Oceanien**  **(n=1)** |
| --- | --- | --- | --- | --- |
| UK (16) | USA (22) | Vietnam (1) | Nigeria (2) | Australia (1) |
| Germany (4) | Canada (3) | Iran (1) |  |  |
| Belgium (1) | Argentina (1) |  |  |  |
| Spain (2) |  |  |  |  |
| Switzerland/France (4) |  |  |  |  |
| Norway (4) |  |  |  |  |
| Finland (2) |  |  |  |  |
| Netherlands (1) |  |  |  |  |
| Greece (2) |  |  |  |  |

**Figure 1 Supplementary: Distribution of studies of PREMs in pediatric healthcare by year of publication**

**Figure 2 Supplementary: Percentage of domain areas**
